# Estimation of heterogeneity in malaria transmission by stochastic modelling of apparent deviations from mass action kinetics

**DOI:** 10.1186/1475-2875-7-12

**Published:** 2008-01-11

**Authors:** Thomas A Smith

**Affiliations:** 1 Swiss Tropical Institute, Socinstrasse 57, Postfach CH-4002, Basel, Switzerland

## Abstract

**Background:**

Quantifying heterogeneity in malaria transmission is a prerequisite for accurate predictive mathematical models, but the variance in field measurements of exposure overestimates true micro-heterogeneity because it is inflated to an uncertain extent by sampling variation. Descriptions of field data also suggest that the rate of *Plasmodium falciparum *infection is not proportional to the intensity of challenge by infectious vectors. This appears to violate the principle of mass action that is implied by malaria biology. Micro-heterogeneity may be the reason for this anomaly. It is proposed that the level of micro-heterogeneity can be estimated from statistical models that estimate the amount of variation in transmission most compatible with a mass-action model for the relationship of infection to exposure.

**Methods:**

The relationship between the entomological inoculation rate (EIR) for falciparum malaria and infection risk was reanalysed using published data for cohorts of children in Saradidi (western Kenya). Infection risk was treated as binomially distributed, and measurement-error (Poisson and negative binomial) models were considered for the EIR. Models were fitted using Bayesian Markov chain Monte Carlo algorithms and model fit compared for models that assume either mass-action kinetics, facilitation, competition or saturation of the infection process with increasing EIR.

**Results:**

The proportion of inocula that resulted in infection in Saradidi was inversely related to the measured intensity of challenge. Models of facilitation showed, therefore, a poor fit to the data. When sampling error in the EIR was neglected, either competition or saturation needed to be incorporated in the model in order to give a good fit. Negative binomial models for the error in exposure could achieve a comparable fit while incorporating the more parsimonious and biologically plausible mass action assumption. Models that assume negative binomial micro-heterogeneity predict lower incidence of infection at a given average exposure than do those assuming exposure to be uniform. The negative binomial model moreover provides an estimate of the variance of the within-cohort distribution of the EIR and hence of within cohort heterogeneity in exposure.

**Conclusion:**

Apparent deviations from mass action kinetics in parasite transmission can arise from spatial and temporal heterogeneity in the inoculation rate, and from imprecision in its measurement. For parasites like *P. falciparum*, where there is no plausible biological rationale for deviations from mass action, this provides a strategy for estimating true levels of heterogeneity, since if mass-action is assumed, the within-population variance in exposure becomes identifiable in cohort studies relating infection to transmission intensity. Statistical analyses relating infection to exposure thus provide a valid general approach for estimating heterogeneity in transmission but only when they incorporate mass action kinetics and shrinkage estimates of exposure. Such analyses make it possible to include realistic levels of heterogeneity in dynamic models that predict the impact of control measures on transmission intensity.

## Background

Heterogeneity in transmission rates of parasitic infections is a major source of uncertainty in predictions of the impact of control measures. In general, heterogeneity decreases the effectiveness of untargeted interventions, and increases the effectiveness of those that are targeted to high transmission groups [1]. In order to make quantitative predictions of the impact of control measures we need models with appropriate allowance for heterogeneity.

For most infectious diseases it is difficult, if not impossible, to directly measure heterogeneity. For instance, *Plasmodium falciparum *malaria transmission is measured by the Entomological Inoculation Rate (EIR), which is the product of the human biting rate (*ma*) (mosquito bites per person per night) and the sporozoite rate (the proportion of mosquitoes carrying the infectious sporozoite stage in their salivary glands, *s*) [2]. Both these quantities can be measured only with difficulty and with considerable sampling error. In particular, *ma *is assessed either by human landing collections, which rely on the assumption that young men deliberately expose themselves to biting have similar exposure to the general population nearby, or more indirectly, by using mosquito traps.

Many studies have recorded human landing rates in different malaria endemic communities, and it is possible to partition the variation observed into spatial, temporal, and observer specific components, and to estimate the effects of housing construction, or other interventions. However there is always a substantial residual variance that cannot be accounted for by any measured factor and because any given individual's exposure can only be assessed using one method at any one time, it is unclear how much of the variation between measurements reflects real heterogeneity in exposure and how much reflects the variability inherent in the sampling methods. A frequently cited estimate is that 20% of the population receive 80% of the exposure [1], but this figure is based on an empirical summary of light trap data from which the true heterogeneity cannot be estimated.

It might be the case that there is less real heterogeneity than the heterogeneity in the measurements, because the variation in measurements is inflated by differences in the way the measuring tools are applied on different occasions (random measurement error) and by sampling variation. Conversely, if there are many replications of the measurements the variance of the true exposures might be much greater than that of the averaged measurements.

One approach to estimating the degree of heterogeneity that has been attempted is to fit models to age-prevalence data as a function of EIR, incorporating a parameter for heterogeneity[3], however this entails fitting rather speculative models for immunity (the fact that such a model fits well does not mean that it is correct[4]). Moreover, it is hard to see how this approach can incorporate the effects of unknown imprecision in EIR measurements.

Analysis of the shape of the relationship between infection incidence and measured exposure provides a strategy for estimating the true level of heterogeneity. Most models of infectious diseases assume that transmission occurs at a rate directly proportional to the number or density of both susceptible and of infectious hosts. This mass action assumption corresponds to the established biology of infection in homogeneous populations, but where transmission is heterogeneous the population average force of infection is no longer proportional to the average density of infectious hosts, or (in the case of vector borne diseases) to the vectorial capacity [5] and a lower proportion of individuals is infected at any given exposure than is predicted by mass action. If the average exposure and the susceptibility of the hosts are known, then the reduction in the force of infection below the mass action expectation can be used to estimate the amount of micro-heterogeneity. A complication in the case of *P. falciparum *is that susceptibility in the field is uncertain, and the imprecision of exposure estimates also needs to be taken into consideration.

This article applies a statistical approach to estimate the level of heterogeneity in *P. falciparum *transmission from exposure-infection relationships in field data from Kenya. The method uses negative binomial models to simultaneously estimate the level of heterogeneity and the susceptibility, incorporating exposure estimates adjusted for effects of sampling variation and assuming underlying mass action kinetics. The estimate of the degree of micro-heterogeneity in transmission provided by this model gives an empirical basis for the level of heterogeneity to incorporate in predictive models. This model is contrasted with other models fitted to the same data that assume either facilitation or competition between different inocula, implying dose-dependence in the infection process [6].

## Methods

### Field data

The study of Beier and others [7] in Saradidi, Kenya, represents the most informative dataset available on the relationship between *P. falciparum *exposure and infection.

In this study, *P. falciparum *parasites were cleared using sulphadoxine-pyrimethamine in 21 cohorts children aged between six months and six years. Follow-up of each of the 21 cohorts started at four week intervals, and infection incidence in each cohort was recorded subsequently in four fortnightly parasitological surveys. Each of these four inter-survey intervals, nested within the cohort, defines one sampling unit, *i *= 1,2....84, characterized by a number of children at risk, a proportion, *h*_*i*_, who became infected, and a true average EIR, *e*_*t*(*i*)_, where *t*(*i*) indicates the fortnight (out of the 44 possible) for which the sampling unit was studied.

The entomological data for estimating each value of *e*_*t*(*i*) _were obtained using weekly all-night human biting collections made inside six houses by six teams [8]. Each of the 44 estimates, z_*t*(*i*)_, corresponding to different values of *t*(*i*), thus used a total of 12 human bait collections from which both *ma *and *s *were calculated and the published EIR values [7] represent the sum of the sporozoite positive mosquitoes captured, scaled to give values in infectious bites per fortnight.

The dataset as published by Beier and others[7], includes the numbers of children infected, and those at risk, from which the proportion of children infected (*h*_*i*_) can be estimated for each sampling unit, together with point estimates of *e*_*t*(*i*) _for each fortnight. The present paper reports a series of statistical models for the relationship between *h*_*i *_and estimates of *e*_*t*(*i*) _made using different models for the distribution of inoculations in the population and mprecision in the EIR measurements.

Patterns of seasonality in the data were reported by Beier and others [8] and the original publication was followed in relating recorded infection incidence to concurrently measured exposure. No details of the sampled houses, of day-to-day variation within each two week period, or of inter observer variation in biting rates are available.

### Models for the relationship of infection incidence to EIR

Children attract fewer mosquitoes than adults [9,10], and hence *e*_*t*(*i*) _is higher than the average number of infectious bites received by children. The Saradidi dataset does not provide any disaggregation of either infection or exposure data by age, and so the extent of this effect must be inferred from other data. In previous analyses[11], average weights of children were used in an East African population to obtain a scale factor, *w *= 0.302, to be applied to the children in the Saradidi study to allow for this effect, so that the average age adjusted EIR value, *e*_*at*(*i*)_, is:

*e*_*at*(*i*) _= *we*_*t*(*i*)_

Let the random variable *x*_*ij *_be the actual number of infectious bites received by individual *j *in unit *i *any one time period, and let *S*_*i *_be the probability that an inoculation in sampling unit *i *survives to give a patent blood-stage infection. Computationally, it is more straightforward to treat the effect of host size as equivalent to a reduction in this survival probability, so that the number of inoculations experienced by children is nominally also *x*_*ij*_, sampled from the same distribution (with mean *e*_*t*(*i*)_) as for adults, while the effective survival of the inoculum in children is:

*S*_*ai *_= *wS*_*i*_

Each child then has probability *h*_*ij *_that it will be infected from at least one of the *x*_*ij *_bites, where, in the general case, assuming independence between the bites, the random variable

$$h_{ij}=1-{\left(1-S_{ai}\right)}^{x_{ij}}.$$

Various models were consider that differ in terms of the functional form of *S*_*i *_as follows:

#### a. Mass Action

The simplest stochastic model for the infection process assumes that *S*_*i *_is a constant, *S*, and hence *S*_*ai *_= *wS*.

#### b. Facilitation

We follow [6] in considering a model where the force of infection is a sigmoidal function of the infectious dose. To ensure that the modelled survival probability of the inoculum, *S*_*ai*_, remains between the limits of 0 and 1 we apply the sigmoidal function to *S*_*ai *_directly, rather than explictly modelling a sigmoidal relationship between *E*(*h*_*ij*_) and *e*_*at*(*i*) _using the Hill function:

$$S_{ai}=\frac{{\left(e_{at(i)}/E_{f}^{*}\right)}^{\gamma }}{1+{\left(e_{at(i)}/E_{f}^{*}\right)}^{\gamma }}\text{ (}\gamma \text{>1)}\text{.}$$

The model for human infection thus has two parameters, $E_{f}^{*}$ and *γ*.

#### c. Saturation

Descriptive analyses of the Saradidi data indicated that the survival probability of the inoculum appears to decrease with increasing transmission intensity [7], suggesting that there is density dependence in the infection probability. In principle, this might arise because of saturation at high inoculation rates, as assumed in the malaria model of the Garki project [12]. [13] adapted the function used in the Garki model and fitted it to the Saradidi data assuming:

*E*(*h*_*ij*_) = *g*(1 - exp(-*βe*_*at*(*i*)_))

where the two parameters *g *and *β *are both measures of susceptibility. This model was generalized by considering uncertainty in *e*_*at*(*i*) _as well as in the proportion of children infected. This leads to different expressions for *S*_*ai *_depending on the distribution of *e*_*at*(*i*) _or equivalently *e*_*t*(*i*) _(see below).

#### d. Competition

Further exploratory analyses of the Saradidi data suggested that saturation does not occur [11], and a model was, therefore, proposed that explicitly captures the decrease in survival of the inoculum with increasing EIR but avoided the assumption of saturation. This was done by making *S *depend on *e*_*at*(*i*)_:

$$S_{ai}=S_{\infty }+\frac{1-S_{\infty }}{1+\frac{e_{at(i)}}{E_{c}^{*}}}$$

The parameter *S*_∞ _is then the lower limit attained by *S*_*ai *_as the inoculation rate becomes large, and $E_{c}^{*}$ is the value of *e*_*at*(*i*) _at which half the reduction in *S*_*ai *_is achieved.

### Models for imprecision in the estimation of the EIR

The assumptions that we make about the distribution of imprecision in the measurements of the EIR are reflected in the distribution used for the number of bites on an individual, *x*_*ij*_, with consequences for the probability that a child is infected.

#### a. No shrinkage of estimates of *e*_*t*(*i*)_

In the first set (models 1–4) following previous analyses of the Saradidi data, we treat the true average EIR for the sampling unit, *e*_*t*(*i*)_, as given by the data, ignoring both variation between individuals in exposure and imprecision in the determination of the average, so that both the individual exposures *x*_*ij*_, and the average EIR, *e*_*t*(*i*)_, are assumed equal to the measured value, *z*_*t*(*i*)_.

Correspondingly the force of infection in children, equal to the intensity of the infection process, is assumed known, as *S*_*ai*_*z*_*t*(*i*)_, and treating the number of these infections per individual as Poisson distributed the probability that a child receives at least one infection is:

*E*(*h*_*ij*_) = 1 - exp (*-S*_*ai*_*z*_*t*(*i*)_)

#### b. Poisson variation in estimates of *e*_*t*(*i*)_

In the second set (models 5–8), recognising that *z*_*t*(*i*) _is obtained by counting sporozoite positive mosquitoes, the count of infections recorded in two weeks per mosquito collector is assumed to be a Poisson distributed random variable with expectation equal to the true average for the sampling unit:

*z*_*t*(*i*) _~ *Poisson*(*e*_*t*(*i*)_)

The expected value of *z*_*t*(*i*) _remains equal to *e*_*t*(*i*)_, but there is random Poisson variation around this expectation.

We again assume that the number of infections per individual, *x*_*ij*_, is Poisson distributed, but the mean of the Poisson is now the unknown quantity *e*_*t*(*i*)_, rather than the observed number of inoculations *z*_*t*(*i*) _The probability that a child receives at least one infection is therefore:

*E*(*h*_*ij*_) = 1 - exp (-*S*_*ai*_*e*_*t*(*i*)_)

In the case of the model of saturation, where equation (5) does not give an explicit expression for *S*_*ai*_, the survival probability of the inoculum is then obtained by combining equations (5) and (9) to give:

$$S_{ai}=\frac{-\ln\left[1-g(1-\exp(-\beta e_{at(i)})\right]}{e_{t(i)}}$$

*e*_*t*(*i*) _is now a vector of parameters to be estimated conditional on the observed values of *z*_*i*_

#### c. Negative binomial variation in estimates of *e*_*t*(*i*)_

Finally (models 9–12), we consider *z*_*t*(*i*) _as negative binomially distributed with mean *e*_*t*(*i*) _and overdispersion parameter *κ *i.e.:

*z*_*t*(*i*) _~ *NB*(*r*, *π*_*t*(*i*)_)

$$E(z_{t(i)})=\frac{r(1-\pi_{t(i)})}{\pi_{t(i)}}=e_{t(i)},$$

where *κ *= 1/*r *and,

$$\mathrm{var}(z_{t(i)})=e_{t(i)}+\kappa e_{t(i)}^{2}.$$

We again assume *x*_*ij *_has the same distribution as *z*_*t*(*i*)_, and apply equation (3). To obtain an approximation for the expected value of *h*_*ij *_(for models 9,10 and 12), we use a second order Taylor expansion about *e*_*t*(*i*) _In general, where *f*(*x*_*ij*_) is some general function of *x*_*ij*_, then its expectation can be approximated with:

$$E(f(x_{ij}))\approx f(E(x_{ij}))+\frac{1}{2}\mathrm{var}(x_{ij}){\left(\frac{d^{2}f(x_{ij})}{dx_{ij}^{2}}\right)}_{x_{ij}=E(x_{ij})}.$$

In the specific example in question:

$$E(h_{ij})\approx 1-{\left(1-S_{ai}\right)}^{e_{t(i)}}-\frac{e_{t(i)}+\kappa e_{t(i)}^{2}}{2}{\left(1-S_{ai}\right)}^{e_{i}}{\left(\ln(1-S_{ai})\right)}^{2}.$$

For the model with saturation (model 11), *E*(*h*_*ij*_), is given explicitly by equation (5).

### Model fitting

Treating *e*_*t*(*i*) _as known (models 1–4) leads to non-linear models with binomial errors in the infection probability, for which maximum likelihood methods could easily be applied. The remaining models represent hierarchical random effects models, which can be fitted most easily using a Bayesian Markov chain Monte Carlo (MCMC) algorithm. For consistency MCMC was used to fit all the models, using WinBugs 1.4 software [14] and treating the proportion of children infected in each sampling unit (i.e. each 14 day period within each cohort) as binomial with probability *E*(*h*_*ij*_). Uniform(0,1) prior distributions were assigned for the proportions *S*, *g*, *S*_∞_, log-normal priors with large variance to $E_{c}^{*}$, $E_{f}^{*}$ and *β*, and a gamma prior for *γ*. For the models with negative binomial variation in the inoculation rate we assign inverse gamma priors to *κ*. Point and interval estimates of all the quantities in equations (4) to (10) were obtained by sampling their posterior distributions. The deviance information criterion (DIC) [15] was used to compare the statistical fit of the models.

## Results

**Figure 1:**
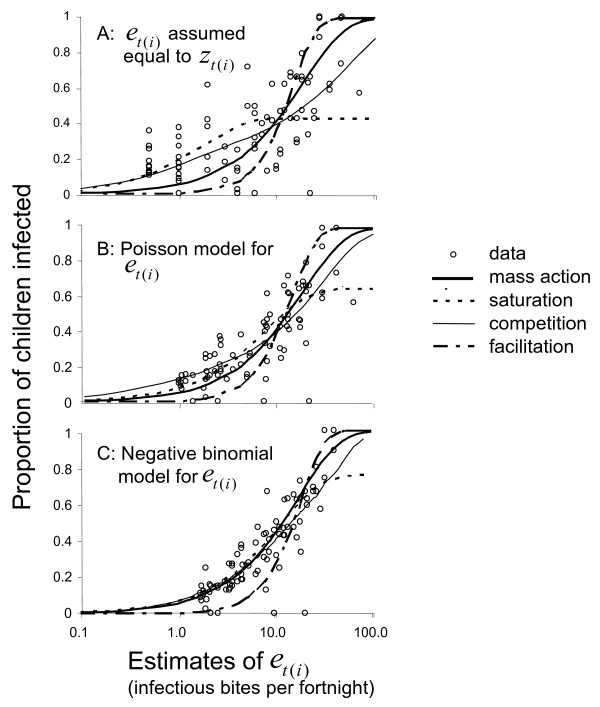
**Model fit and predictions**. The vertical scale corresponds to fitted values of *E*(*h*_*ij*_) for the lines, and the observed proportions for the data points. The horizontal scale corresponds to the shrinkage estimates of *e*_*t*(*i*) _for the Poisson and negative binomial models (assuming competition), and to the original untransformed *e *values for the models where this quantity is assumed known. Note that a few points are superimposed.

Figure 1A give the proportions of children infected plotted against the measured EIR, *z*_*t*(*i*)_, for each of the 83 sampling units where at least one child was at risk at the start of the fortnight. At the start of follow-up the numbers of children in each cohort ranged between 43 and 50, but the numbers at risk decreased incrementally as children were infected and thus censored from the analyses. Overall 1,999 periods at risk were analysed and 617 (30.9%) of these ended with an infection.

Initial analyses considered whether these 83 sampling units can be considered as statistically independent. At the time of the study, parasitological resistance to sulphadoxine-pyrimethamine was extremely infrequent in Kenya, so it is assumed that this treatment cleared all pre-existing infections, and hence there should be no carry-over effects from previous infections. The children with parasites at the start of each period of follow-up were were not included in the risk set for subsequent time points, so no more than one infection event was recorded for any one individual, and the successive observations are statistically independent. However, it remained *a priori *possible that the relative risk of infection in a cohort might decrease over time, because the more susceptible children will be infected (and censored from the cohort) sooner. This possiblity was evaluated by using logistic regression to compare the risk of infection (*h*_*i*_) during the 3^rd ^and 4^th ^fortnights of follow-up, with that during the 1^st ^and 2^nd ^fortnights respectively in the parallel cohorts that started follow-up 4 weeks later. This analysis gave an odds ratio of 1.25 (95% confidence intervals 0.98, 1.59), suggesting that if anything, infection rates tend to be higher in the latter part of the follow-up, thus justifying treating the different cohorts and sampling periods as statistically independent in the subsequent analyses.

As previously reported [7,11,13], the incidence of infection in the Saradidi dataset does not increase in proportion to the measured inoculation rate (Figure 1). As a consequence, the mass action curve fits poorly in models that treat the EIR as known (Figure 1; Table 1). At low EIR values, the best-fitting mass action model (model 1), which estimates that 18% of the inocula result in infection, predicts infection rates much lower than those observed. Facilitation (model 2) fits even less well (Table 1), while saturation (model 3) is a considerable improvement, but shows a poor fit at high EIR, estimating a value of *g *= 0.43, implying that even at the highest EIR only 43% of children would be infected. Competition (model 4) fits best among those treating EIR as known, corresponding to a previously reported result[11]. In addition to these analyses, analyses assuming a two-week lag between inoculation and parasitaemia were also carried out, but the fit of these models was poorer than that of original model.

**Table 1:** Summary of model fit

	Model for imprecision in inoculation rate	Model for relationship of infection to inoculation rate	Deviance Information Criterion		
			Inoculation rate measurement	Infection of children	Total
1	none	Mass Action	-	772.7	-
2	none	Facilitation	-	1840.3	-
3	none	Saturation	-	528.1	-
4	none	Competition	-	486.9	-
5	Poisson	Mass Action	270.2	392.1	662.3
6	Poisson	Facilitation	356.7	411.9	768.6
7	Poisson	Saturation	252.8	383.6	**636.4**
8	Poisson	Competition	**245.8**	392.5	638.3
9	negative binomial	Mass Action	267.4	373.5	640.9
10	negative binomial	Facilitation	277.5	376.3	653.8
11	negative binomial	Saturation	268.8	**371.8**	640.8
12	negative binomial	Competition	266.8	373.9	640.7

The best fitting models are indicated in bold.

**Figure 2:**
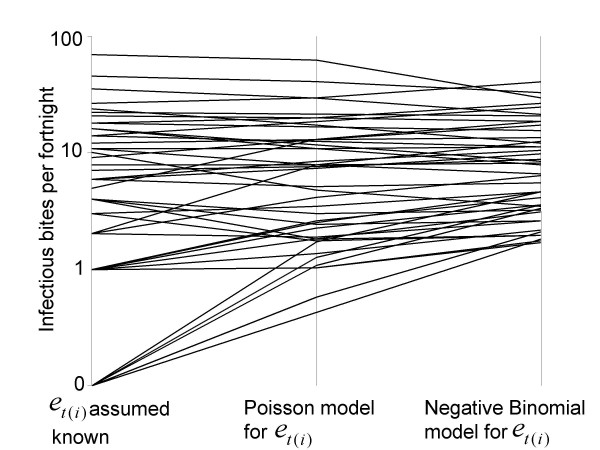
**Effect of model of variance on estimates of *e*_*t*(*i*)_**. Each line corresponds to the different estimates of *e*_*t*(*i*) _made for one fortnight (competition models). Estimates of *e*_*t*(*i*) _for the mass action, facilitation, and saturation models were similar to those for competition models.

Hierarchical models that allow for Poisson variation in the EIR explanatory variable, as well as binomial variation in infection probability, lead to shrinkage of the estimated EIR values towards the mean, thus the points in Figure 1b are much closer together horizontally than are those in Figure 1a. This is illustrated by the triangular shape of the plot of how the estimates of *e*_*t*(*i*) _change as the models allow for more statistical error (Figure 2). Counter-intuitively therefore, the more heterogeneity is assigned to the processes that generated the data, the less heterogeneity between the EIR estimates for the different sampling units.

At high EIR *e*_*i *_is determined more precisely, so there is less effect of the shrinkage in the EIR estimates at high *e*_*t*(*i*) _than at low *e*_*t*(*i*)_. Corresponding to this, the fitted curves relating infection rates to these EIR values are much more similar in Figure 1b (models 5–8) than in Figure 1a. Mass action (model 5) now estimates that 23% of inoculations result in infections. The deviance information criteria (DIC) for these models comprise two terms, one measuring the fit to the EIR data, and one to the data for infection. All four DIC for the fit to the infection data are lower than the value for the best fitting model without the Poisson error term, with saturation (model 7) fitting best among those including Poisson variation in EIR (Table 1), estimating an upper limit of 65% of children becoming infected. The best fit to the EIR data is achieved with competition (model 8), but overall this model has a slightly inferior fit to that of saturation (model 7).

When negative binomial variation in the EIR is assumed, all of the models 9–12 estimate similar degrees of overdispersion, and the estimated underlying EIR values are even more tightly distributed around the average (compare Figure 1c with Figure 1b). A consequence is that the fitted curves for different variations from mass action are also closer to each other than the curves shown in either Figures 1a or 1b. In the range of 1–10 infectious bites per fortnight, into which most of the estimates of EIR fall, the curves for mass action, saturation and competition models are almost superimposed. Over this range of EIR only the facilitation model gives a markedly different estimate of the relationship between infection and EIR, predicting lower infection rates that those observed.

The DIC values for the different negative binomial models are also similar to each other, though facilitation (model 10) again fits markedly worse than mass action (model 9), saturation (model 11), or competition (model 12). Saturation (model 11) now has the best fit to the infection rates (as measured by the DIC), though the level at which saturation occurs is almost the same whether Poisson or negative binomial errors for the EIR are assumed. However, the overall best fit among negative binomial models is achieved assuming competition (model 12), with the mass action only a little inferior. When all twelve models are compared, saturation with Poisson errors (model 7) gives the best fit, as assessed by the DIC (which penalizes the negative binomial models for the extra variance parameter estimated). From a statistical point of view, the negative binomial model is more complicated and the trade off between improvement in fit and complexity of the model tends to speak in favour of a Poisson model.

**Figure 3:**
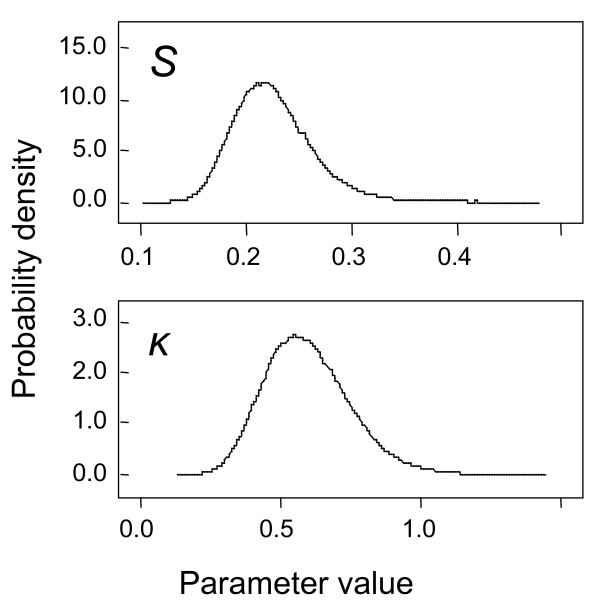
**Posterior distributions of parameters of the negative binomial mass action model**. Estimates based on samples of 100,000 values for each parameter.

Although the Deviance Information Criterion did not indicate a preference for the negative binomial models over Poisson saturation or competition models, the best estimates of *κ *were clearly greater than zero, indicating significant over-dispersion (Table 2; Figure 3). Biologically, models incorporating overdispersion of parasite transmission are much more plausible than Poisson models, since there are many known sources of variation in host exposure that are expected to lead to overdispersion. Plots of the risk of infection as a function of *κ *(not shown) confirm that mass action models with higher levels of variance in the inoculation rate lead to similar predictions to those of saturation or competition models with less variability in *e*_*t*(*i*)_.

**Table 2:** Parameter values measuring overdispersion of the negative binomial models.

Model	κ	95% interval	r	(95% interval)
Mass Action	0.60	(0.34, 0.93)	1.78	(1.08, 2.95)
Facilitation	0.84	(0.56, 1.16)	1.23	(0.86, 1.77)
Saturation	0.62	(0.31, 1.10)	1.78	(1.68, 3.22)
Competition	0.58	(0.32, 0.90)	1.85	(1.11, 3.09)

**Figure 4:**
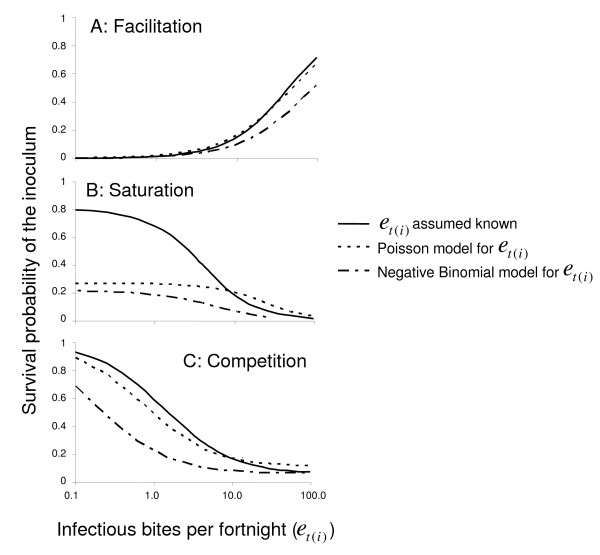
**Predicted survival of inocula (*S*_*i*_)**. The estimated value of *S*_*i *_is plotted against the estimate of *e*_*t*(*i*) _for each model (excluding mass action models, for which *S*_*i *_is a constant (Table 3)).

The plausibility of the different models also depends on the shape of the relationship between inoculum survival, *S*_*i*_, and the EIR (Figure 4). A constant value of *S*_*i *_(i.e. a mass action model) is highly plausible. Competition and saturation models are less parsimonious because they imply that different inoculations interact, even in situations where there are no established blood stage infections and where none of the incoming infections result in blood stage infection. As well as having the poorest fit, the facilitation model leads to the least plausible patterns in the estimates of *S*_*i*_, as a result of the constraint that in the limit, as *e*_*t*(*i*) _→ 0, *S*_*i *_= 0. It would be possible to fit alternative models assuming other limiting values of *S*_*i *_but this would introduce an additional element of arbitrariness into the models.

In the competition model, the more sampling error is assumed in the EIR, the lower is the estimated success probability *S*_*i *_at given EIR (Figure 4). This is because the relatively high *S*_*i *_at low measured EIR is now attributed to sampling error in the EIR measurements. The same effect is observed in comparing the saturation model without heterogeneity with the corresponding Poisson model.

**Table 3:** Estimated parameter values

Model for inoculum survival		Units	No imprecision in inoculation rate		Poisson imprecision in inoculation rate		Negative binomial imprecision in inoculation rate	
			point estimate	95% interval	point estimate	95% interval	point estimate	95% interval
mass action	*S*	proportion	0.18	0.15, 0.20	0.18	0.15, 0.20	0.23	0.17, 0.31
facilitation	$E_{f}^{*}$	inoculations/fortnight	13.4	11.8, 15.1	15.1	11.6, 19.2	27.2	10.7, 52.8
facilitation	γ	dimension-less	1.17	1.00, 1.60	1.02	1.00, 1.08	1.00	1.00, 1.01
saturation	*g*	proportion	0.43	0.39, 0.47	0.65	0.56, 0.77	0.76	0.61, 0.96
saturation	β	fortnights/inoculation	1.89	1.40, 2.51	0.42	0.28, 0.62	0.29	0.17, 0.45
competition	$E_{c}^{*}$	inoculations/fortnight	0.40	0.30, 0.52	0.22	0.10, 0.39	0.01	0.00, 0.03
competition	*S*_*inf*_	proportion	0.06	0.03, 0.08	0.11	0.07, 0.15	0.06	0.04, 0.09

## Discussion

Micro-heterogeneity in exposure to infectious mosquito bites contributes to sampling variation in estimates of the average exposure to *P. falciparum *and hence inflates the variation between exposure estimates for different time periods. It also modifies observed relationships between infection rates and average exposure, because heterogeneous exposures reduce the proportion of individuals that get infected. This effect, the result of superinfections occuring disproportionately in some individuals, becomes greater as exposure increases.

The analyses of this paper compare different distributional assumptions for the sampling variation that contributed to the observed average time-period specific exposures and make shrinkage estimates of the true average exposures that allow for this sampling variation.

The same distributions of entomological inoculations around the population average are used to infer the dependence of the probability of infection on the average EIR. By assuming mass action kinetics and taking advantage of the availability of both infection and exposure data from the Saradidi site, the models provides estimates of the true degree of micro-heterogeneity that best accounts for observed infection-exposure relationship.

Mass action kinetics must apply at the level of individual host parasite interactions but field data on the kinetics of *P. falciparum *transmission from the mosquito vector to the human host seem at first sight to be incompatible with this. This is not a consequence of facilitation, rather the risk for a human of becoming infected for each sporozoite positive mosquito bite appears to decrease steeply with increased transmission intensity. This result is confirmed by both studies of highly exposed cohorts[7,16], and comparisons of the force of infection between laboratory challenge, low transmission areas, and high transmission areas.

Although epidemiological evidence suggests that acquired immunity offers little or no protection against pre-erythrocytic stages of the parasite in children[11] apparent competition between inocula could result from immunological cross reactions. In particular, highly exposed individuals are likely to have been more highly exposed in the past and so may have reduced levels of detection of parasites, which could lead to an overall bias in estimates of *S*. But in the present dataset, the exposure variation arises as a result of short-term temporal variation, and the cohorts are sampled from a common age group in the same population, so acquired immunity cannot account for differences between sampling units.

Innate immunity does seem to play a role in limiting super-infections of *P. falciparum *at the hepatic stage. In particular, asexual parasites stimulate production of interferon-*γ*, which leads to killing of infected hepatocytes[17,18]. Some malaria models, such as those of [12] and [11] have used deterministic functions to capture such apparent density dependent regulation of the inoculation rate. However only if the host is already infected does it mount any of these defensive responses. Feedback from pre-existing infections cannot explain the apparent density dependence in the Saradidi dataset, where all the asexual parasites were cleared at the start of the follow-up.

The known biology of malaria thus gives us no reason to suppose that there is any dependence in the infection probability for successive parasites challenging a given uninfected host, and hence all attempts to model deviations from mass action by treating the success probability of the parasite as an explicit function of the intensity of the parasitological challenge introduce a divorce between the mathematical model and the biology of the infection process. Heterogeneities in transmission therefore provide a more parsimonious explanation for apparent competition than do density-dependent host responses. In sub-populations with higher contact rates the probability of becoming infected is higher, but the increase is not proportional to the contact rate because there is a higher chance of an infection being 'wasted' on a host that has already been colonized, so that heterogeneity mimics the effect of density dependence on infection risk.

The present analyses show that models of heterogeneity can fit field data as well as models of density dependence. Humans are known to be heterogeneous in their attractiveness to mosquitoes[19,20] even in standardized environments. In the field setting, heterogeneities are to be expected both in the exposure and susceptibility of the children within any one cohort and in the sampling efficiency of the different mosquito collectors.

Quantifying focality in transmission via the negative binomial provides a parsimonious model for this micro-heterogeneity that readily accounts for the deviations from linearity in Figure 1A, and gives an estimate of *κ *that is otherwise unobtainable. Although the dataset is the best available with which to estimate of *κ *by this approach, the study was not designed with this purpose in mind and some approximations were required. The most straightforward field design to estimate the level of micro-heterogeneity would have involved estimating the EIR for each fortnight by sampling a single random individual over all 14 nights, corresponding directly to a sample from *z*_*t*(*i*)_. The data were treated as though they had been generated this way by applying the same estimate of *κ *= 0.6 (for the mass action model in Table 2) both to the distribution of fortnightly exposure in children, translated into the formula for infection risk via equation (15), and to the distribution of fortnightly EIR estimates compiled by summing 12 distinct nightly human landing collections (and rescaling them). In the actual design, the employment of 6 different mosquito collectors rather than one only, presumably brought the overall EIR estimates closer to the population average. Conversely the shorter sampling periods, involving sampling only 12 of the 14 nights during each fortnight, leads to inflation of the variance of the EIR estimate relative to that in the ideal design. These two effects appear to have approximately cancelled each other out: when the negative binomial mass action model was extended to allow distinct values of *κ *for the sampling of the EIR and for the formula for infection risk, both *κ *estimates were close to the original value of 0.6, with the difference between them only 0.06 (95% CL for the difference: -0.57, 0.65).

The Saradidi environment can be assumed to be reasonably typical of rural East Africa, and the estimation of *κ *represents a critical step in parameterising mathematical models for heterogeneity in malaria transmission in such environments. The next step, of partitioning the variance into variation in space, variation between different human hosts, and between different nights during the fortnight, requires data from other sites.

A complete model for heterogeneity in malaria transmission also needs to consider transmission from human to vector. This also seems to defy mass action. Detailed records of both gametocyte densities and transmissibility to vectors are available from neurosyphilis patients who received therapeutic malaria infections prior to the availability of antibiotics. Higher asexual parasite densities are associated with higher gametocyte densities, and these in turn are associated with greater transmission to the vector [21], but the force of infection of the vector does not increase in proportion to host parasite densities, even allowing for the need of oogenesis for gametocytes of both sexes. A stochastic model was recently fitted to these data that accounts for the observed lack of proportionality as a consequence of random variation in gametocytogenesis[22]. In conjunction with the estimate of *κ *= 0.6 for vector-human transmission, this makes it possible to use field based estimates of heterogeneity in models of the whole *P. falciparum *transmission cycle.

## Conclusion

The present study presents a method for estimating the extent of micro-heterogeneity in exposure to infected malaria vectors, at the same time demonstrating that heterogeneity can explain apparent deviations from mass action kinetics. The estimates obtained can be used in formulating dynamic models of the whole malaria transmission cycle, in which stochastic variations in transmission rates are used to mimic the actual levels of heterogeneity found in the field. This will make it possible to model the impact of malaria control interventions while allowing for realistic levels of heterogeneity in transmission.

## Competing interests

The author(s) declare that they have no competing interests.

## Authors' contributions

The author alone carried out the data analysis, writing, and revision of the manuscript.
